# Repercussions of the Cross-Border Migration Process on Family Life: Systematic Review with Meta-Synthesis

**DOI:** 10.3390/ijerph23020165

**Published:** 2026-01-28

**Authors:** Mateus Souza da Luz, Vanessa Bordin, Sonia Silva Marcon, Gabriel Zanin Sanguino, María José Cáceres-Titos, Chang Su, Mayckel da Silva Barreto

**Affiliations:** 1 Postgraduate Program in Biosciences, State University of Western Paraná, Cascavel 85819-110, PR, Brazil; 2 Department of Nursing, State University of Maringá, Maringá 87020-900, PR, Brazil; 3 Department of Nursing, Huelva University, 21071 Huelva, Spain; 4 Department of Psychology, Brandon University, Brandon, MB R7A6A9, Canada

**Keywords:** family care, migrant health, literature review, public health nursing, health

## Abstract

**Highlights:**

**Public health relevance—How does this work relate to a public health issue?**

- Examines how cross-border migration reshapes family functioning, mental health, and social well-being—core components of population health.
- Highlights the structural vulnerabilities experienced by migrant families, including poverty, isolation, and limited access to essential services.

**Public health significance—Why is this work of significance to public health?**

- Provides synthesized evidence on how migration-related stressors contribute to psychological distress, family conflict, and risk behaviors among migrants.
- Offers critical insights into the social determinants of health that disproportionately affect migrant families across diverse global contexts.

**Public health implications—What are the key implications or messages for practitioners, policy makers and/or researchers in public health?**

- Emphasizes the need for culturally sensitive health, social, and community interventions to support migrant family adaptation and mental well-being.
- Informs policy development aimed at reducing social vulnerability, improving service accessibility, and strengthening protective family and community networks.

**Abstract:**

The experiences and repercussions of the cross-border migration process on family life have not yet been synthesized. This study aimed to synthesize the best available qualitative findings on this theme. A systematic review of qualitative evidence with meta-synthesis was conducted. Articles were identified according to predefined extraction criteria in the first half of 2025, across seven databases: Web of Science, MEDLINE/PubMed, PsycINFO, LILACS, CINAHL, SCOPUS, and Social Science Citation Index. Two researchers independently screened and appraised the reports, assessing methodological quality and systematically recording and analyzing relevant information. A protocol was registered in PROSPERO (ID: CRD42024505655). Fifty studies were included, and three main themes emerged: (a) living in multiple possible contexts, where space and relationships influence family functionality, including reduced family time due to long working hours, substance use, fear of losing cultural roots, new financial responsibilities, and the desire to return to the country of origin; (b) challenges and repercussions on family life after migration, such as increased family conflicts, mental health problems, separation, and loss of ties; (c) strategies for maintaining family functioning, including role adjustment, strengthening of family ties, and support through cultural and religious practices. Families undergoing migration face multiple challenges in their new environments, revealing the complexity of adapting to diverse cultural and social contexts. These findings highlight the need to address the emotional and social demands of migrant families to improve well-being and integration. Understanding these dynamics allows healthcare professionals to design culturally sensitive interventions that promote reception and inclusion.

## 1. Introduction

Recent statistics indicate that there are approximately 281 million international migrants worldwide, making up about 3.6% of the global population [1]. This phenomenon is driven by a diverse combination of factors, including global consumerism, advancements in transportation, armed conflicts, political repression, economic underdevelopment, violence, poverty, food insecurity, and climate disasters [2]. Additionally, the transformative effects of the COVID-19 pandemic shaped migration patterns [1]. Mobility also reflects the search for better living conditions and safety, driving individuals and families to cross borders to find new opportunities for progress [3,4].

Migration has a significant impact on the social, economic, educational, and health spheres in the host country, as well as personal and family life [5].

Although the reasons for migration are often linked to suffering and the need to seek protection, migrants also grapple with grief from losing their homes and severing ties with their social and family networks [6]. In this context, they may face challenges on their mental health and family dynamics, as adapting to a new reality is not always easy [7].

These difficulties are exacerbated by the challenges and conflicts experienced in the country of origin, and the absence of significant family members left behind. Thus, distancing oneself from the family of origin or the family one has formed can aggravate suffering. Furthermore, the families who remain behind, now missing one of their members, also encounter challenges. Therefore, both migrants and their families may also encounter challenges that require community support and attention from health and social care professionals to identify and implement effective coping strategies [7,8].

In addition, it is important to consider that when families migrate together, they must adapt to new experiences in a challenging environment. This necessity makes cultural adaptations indispensable for individuals and family systems in the destination country. Migration involves not only geographical relocation but also a process of redefining roles and relationships in a new setting, as well as navigating different cultures and worldviews, which can alter family dynamics [8].

Studies conducted in various countries, such as Peru [9], the Netherlands [10], Australia [11], and Italy [12], have deepened our understanding of the complex life and health situations faced by migrants and refugees during continuous adaptation processes, particularly from an individual perspective. Furthermore, in recent years, the experiences of international migrants and their families have been explored through qualitative research [13,14,15,16], leading to the production of knowledge that needs to be synthesized to further deepen understanding of this phenomenon. It is essential to comprehend the experiences of migrants and their families in order to promote new forms of transnational communication among family members, support family adaptation, identify strategies to facilitate reunification, understand their rights, and ultimately foster family integration into the host society [3]. Moreover, developing this understanding can contribute to formulating public and social policies that encourage the restructuring of family systems, improve adaptability to change, and mitigate the biopsychosocial risks encountered by migrant individuals and families.

In light of the above, this review aims to synthesize the best available qualitative findings about the experiences and repercussions of the cross-border migration process on family life.

## 2. Materials and Methods

This study is a systematic review that adopted the JBI meta-synthesis methodology [17]. Additionally, the Preferred Reporting Items for Systematic Reviews and Meta-Analysis (PRISMA) instrument [18] (Supplementary Materials File S1) was used for the writing and detailing of the study. The review protocol was registered in PROSPERO (ID: CRD42024505655) and published in a peer-reviewed journal in the field [19].

The review question was: What are the repercussions of the cross-border migration process on family life? The study was conducted using the PICo strategy (Population; Phenomenon of Interest and Context).

**Population**

Qualitative studies that involved people and families affected by the cross-border migration process and its repercussions were included. Participants comprised both voluntary migrants those seeking better socio-economic and educational opportunities and involuntary migrants who were forced to be displaced due to environmental, racial, economic, social, political, religious, or cultural factors.

**Phenomena of interest**

Studies that have examined the repercussions of the cross-border migration process on families may include family studies conducted with a single family member or those involving at least two members of the same family.

**Context**

Research conducted worldwide has documented the experiences of individuals who have migrated beyond their country’s borders in various contexts. This includes studies focusing on those who migrated alone, in family dyads, or as a part of a collective.

### 2.1. Types of Studies

Studies published between 2009 and 2024 were included, as this has seen a rise in publications addressing the migration process worldwide. The goal was to ensure the review of the most recent literature on the topic. Additionally, only full-text articles published in Portuguese, English, and Spanish were considered, as the researchers are fluent in these three languages and the available resources prevented them from commissioning full translations of articles in other languages.

Only studies that presented qualitative data on the experiences of people and families affected by the cross-border migration process were analyzed. This included, but was not limited to, approaches such as phenomenology, grounded theory, ethnography, action research, and descriptive studies using content, discourse, or thematic analysis. Finally, it should be noted that only studies published in peer-reviewed scientific journals were considered, along with unpublished sources and grey literature such as dissertations and theses.

### 2.2. Search Strategy

The search was conducted between March and May 2025. A preliminary search was performed in the PubMed database to identify relevant articles related to the topic and to validate the initial search strategy. A librarian was consulted to promote rigor in the process. A total of seven databases were used: Web of Science, Medical Literature Analysis and Retrieval System Online (MEDLINE-PubMed); PsycINFO (American Psychological Association), Latin American and Caribbean Health Sciences Literature (LILACS), Cumulative Index to Nursing and Allied Health (CINAHL), SCOPUS, and Social Science Citation Index. However, no articles were found in the last database. The search strategy, including all identified keywords, was adapted for each database according to its specific characteristics.

It should be noted that, following the precepts of the JBI guidelines, the same descriptors were used to search for five sources of unpublished and grey literature, including Index to Theses, Digital Dissertations, CAPES Bank of Dissertations and Theses, Networked Digital Library of Theses and Dissertations, and the New York Academy of Medicine Grey Literature Report. However, no material addressing the review question was identified. Details of the search strategy are provided in the Supplementary Materials (Supplementary Materials Table S1).

### 2.3. Selection of Studies

After the search, all the studies were analyzed in terms of title and abstract, with duplicates removed at this stage. Two team members reviewed the titles and abstracts independently, applying the inclusion and exclusion criteria. Any discrepancies in the selection process were resolved through discussion with a senior researcher in the group, to reach a consensus.

### 2.4. Evaluation of Methodological Quality

The eligible studies were manually assessed for methodological quality by two independent reviewers using the Critical Appraisal Checklist for Interpretive and Critical Research (QARI) [13]. Following this initial assessment, the article documents and the checklist were entered into an Artificial Intelligence (AI) platform, ChatGPT^®^ (version 4.0, OpenAI, San Francisco, CA, USA), with a request for analysis using the following command: “I request analysis of the article using the QARI instrument also attached.” Complete compatibility was observed between the analyses conducted by the researchers and those generated by the AI. However, caution is recommended when using this tool for the same purpose, and its use should be explicitly reported in future studies to enable verification of the compatibility between researchers’ analyses and those produced by AI.

In the quality assessment, articles that did not achieve 70% or more positive responses in the instrument were excluded, which happened in two cases.

### 2.5. Data Extraction and Synthesis

This stage of analyses took place between June and July 2025. Two reviewers independently read and reviewed the articles to systematically extract and record data. They used the Qualitative Data Extraction Instrument, developed by the JBI, which contained the following information: title, authors, year of publication, journal, methodology, data analysis techniques, setting, geographical and cultural context, participants, interventions, main results, and conclusions of both the authors and reviewers. After data extracting, the results were synthesized, representing an aggregation of the primary data. It is noteworthy that all included studies presented the necessary elements for data extraction; in other words, no missing data hindered the completion of the review.

Following the JBI guidelines, the findings were categorized into sub-themes based on descriptive and conceptual similarity, which were then aggregated into broader, more comprehensive themes. To enhance the rigor of the aggregative synthesis process, any differences were discussed by the group until a consensus was reached. Examining the similarities and differences in the perspectives of various social actors who experienced the phenomenon under investigation, sub-themes, and themes were integrated rather than compared. This led to the identification of the meta-theme “Tending between change and maintenance: the search for family functionality in the face of the cross-border migration process”.

## 3. Results

**Figure 1:**
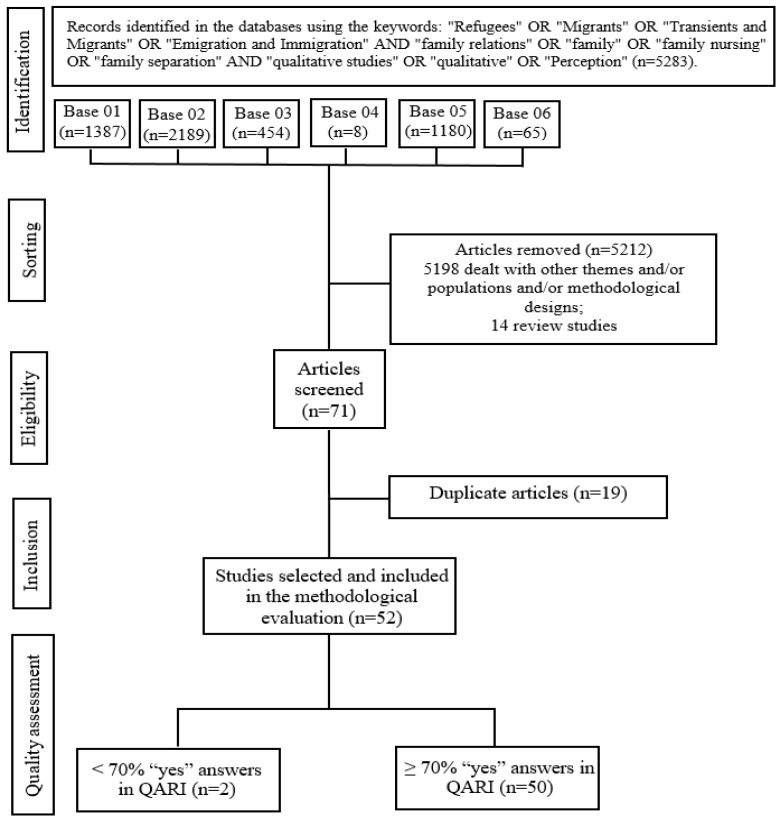
Flowchart for the identification, selection, and inclusion of meta-synthesis studies—Maringá, PR, Brazil, 2025. Source: Authors’ data collection and analysis. Key: Base 01: Web of Science; Base 02: SCOPUS; Base 03: MEDLINE/PubMed; Base 04: LILACS; Base 05: PsycINFO; Base 06: CINAHL.

This review included 50 articles that highlighted the repercussions that the cross-border migration process has on family life. The process of identifying the articles can be seen in Figure 1. 

The studies were conducted in different countries on five continents. The research involved 1995 migrants (Table 1).

### 3.1. Meta-Theme: Tending Between Change and Maintenance: The Search for Family Functionality in the Face of Cross-Border Migration

It was possible to identify that families experiencing the migration process appear to be enmeshed in a metaphorical rope that holds the family system together, despite the individual members possessing distinct perceptions of the situation. This dynamic can be likened to a game of “tug-of-war”, in which family members exert opposing forces on either end. On one side lies the necessity for adaptation, acculturation, and integration into the host country, while on the other resides the desire to maintain their cultural roots, traditions, cultural norms, beliefs, and habits. The different social contexts in which the families find themselves in the new location play a significant role in tilting this balance, causing them to experience greater or lesser repercussions. As a result, strategies are devised to ensure that family systems function properly (Table 2).

#### 3.1.1. Living in Multiple Possible Contexts: Space and Relationships Influencing Family Life

This theme includes the various contexts in which international migrant families find themselves upon settling in a new country. The contexts are influenced by factors such as the place of reception, living conditions, work, access to health, and education. In this sense, some migrants describe living in an underworld marked by poverty, unemployment, insecurity, isolation, xenophobia, discrimination, and a total lack of family and social support. Conversely, some migrants feel lucky to be in a context where there is a higher quality of life due to access to goods and services that were unavailable in their country of origin. It is in this multiplicity of culturally diverse contexts that the lives of the international migrant families are shaped, transformed, and experienced.

##### Living in the Underworld: Experiences of the Precariousness of Life in a Cultural Context

It has emerged that many families settle in host countries and live in an underworld, having to deal with precarious living conditions [13,25,26,32,48,50,55,56,58,59,63]. Many lack access to essential needs such as nutritious food, adequate housing, education, employment, leisure, health, and quality of life. In some cases, they experience food insecurity, resorting to scavenging from waste dumps, and may share their living spaces with animals and vectors, putting their health at risk [50]. This situation is further exacerbated by unemployment or underemployment [41,47], as migrants frequently encounter challenges in securing employment that aligns with their qualifications acquired by their countries of origin, leading them to accept manual labor and informal jobs.

The context in which international migrant families operate is characterized by a notable lack of social support [29], particularly concerning familial support [16,43]. As a result, some migrants report significant social isolation, associated with xenophobia and discrimination. This isolation can arise from various factors, including language and cultural barriers, as well as the absence of proper documentation [16,29] and stable employment opportunities [16,28,29,37,47,50,52]. Additionally, some migrants express feelings of estrangement within society, stemming from communication barriers or their phenotypical and cultural characteristics originating from their home countries [50,52].

Cultural differences have been identified as part of the new context of precarious family life, particularly concerning child-rearing practices. As families encounter new rules, traditions, and cultures, they realize that the cultural mix induces the formation of new cultural traits and rearrangements, which affect the maintenance/propagation of family customs [47].

##### Feeling Lucky to Experience Life with Security, Work, Housing, and Quality

International migrants highlight that a favorable context for living in the host country includes three basic elements: family and community support, social security, and conditions form maintaining quality of life [21,24,25,28,31,32,33,35,37,44,53,55,57,58,63]. The arrival of additional family members increases the sense of support, while the reunification of couples underscores the significance of familial bonds, particularly for children who, in numerous instances, have not had the opportunity to reacquaint themselves with one or both parents or other close relatives. These reunions promote meaningful family moments filled with leisure, play, and interaction. Family life and support are widely recognized as fundamental elements for quality of life [35].

Migration to North American or European countries, for instance, has been identified as providing families with a heightened sense of security within the social environment when carrying out daily activities, such as grocery shopping [51]. This aspect is regarded as a positive aspect of the new context, as family security, satisfaction with the host country, and a vision of the future, especially for raising children, were identified as criteria that synergistically come together to facilitate the process of adaptation and integration into the new country [44,48,53].

The new living conditions in the host country are mentioned as positive aspects of the process of adaptation, acculturation, and integration, especially the quality of housing and access to decent and profitable employment. In addition, conditions are limited in the country of origin, and there is no possibility of access to the material goods they enjoyed at the time [21,24,32].

#### 3.1.2. Challenges Faced and Repercussions on Family Life After Migration

The analysis reveals that family life is significantly influenced by the myriad challenges and confrontations experienced by migrants. In many cases, family life is shorter/limited due to long working hours, so they have to deal with greater conflicts and changes in mental health, face separation and homesickness. In addition, families have to deal with the abuse of licit and illicit drugs; greater financial responsibilities, including for those who stayed in the country of origin; and the fear of losing their cultural roots in the new context. This complex scenario ultimately contributes to a pronounced desire to return to their country of origin.

##### Potentiating Family Conflicts

Changes in family dynamics directly influence interpersonal relationships, structure, and functionality, requiring constant adaptation to deal with the challenges imposed by migration. In many cases, this process exacerbates stress due to the vulnerability imposed, leading to arguments between parents and children [16,25,26,27,28,29,30] and domestic violence and divorce between spouses [13,26,27,30,31,49,52]. Thus, migration can heighten inequalities and emotional challenges in families, requiring continuous adaptation in the face of often adverse circumstances.

##### Repercussions on Mental Health

It has been shown that the migration process has a profound impact on people’s emotions, exacerbating feelings of loneliness, sadness, and anxiety [13,21,41,45,48,55,59,63] and, in extreme cases, leading to suicidal thoughts [21,45]. Family separation and estrangement are the main causes of suffering and anguish [21,28,41,44,48]. In this way, migration can cause intense psychological changes, profoundly affecting the mental and emotional health of migrants, who often face social isolation and distance from their support networks.

##### Family Separation, Loss of Ties, and Homesickness

Separation and the loss of ties, resulting from the physical and emotional estrangement of the members who stayed [24,27,28,31,41,54,61] or forced separation during the migration process [21,25] often lead to disconnection that profoundly alters family dynamics and has both psychological and emotional repercussions for the individuals. When reunions take place after years of separation, individuals may experience feelings of indifference, distance, rejection, and resentment [21,41,46,52,54]. This disruption illustrates how migration can cause lasting damage to family relationships, resulting in irreparable losses. On the other hand, for many migrants, the emotional and physical distance from people with whom they had strong ties, such as grandparents and parents, and the changes in family dynamics, such as the loss of the possibility of having dinner together and talking, generate feelings of emptiness and loneliness [21,26,29,36,37,41,51,52,53].

##### Reduced Family Life

Even when an entire family migrates together, it often disrupts family life, daily routine, and family dynamics. Many migrants report challenges in balancing long working hours with family life [47,49]. This lack of coexistence is intensified by the overload of domestic responsibilities that end up falling on women, with double or triple working hours, such as looking after the house and the children and still working [49]. This change takes parents away from everyday activities and weakens the family bond that used to be sustained by time spent together.

##### Use of Psychoactive Substances

The use of psychoactive substances after migration is a challenge that affects family dynamics. The biggest problem is that Alcohol consumption by men, who are almost always the main breadwinners, is particularly concerning, as it not only strains family finances but also has profound emotional impacts [25,48,63]. These situations show how the consumption of alcohol and other drugs among migrants can destabilize family finances and create tensions in the home, exacerbating the already difficult adaptation to the new reality.

##### Fear of Losing Cultural Roots

The fear of losing cultural roots after migration is a common concern among many migrants, who face the challenge of preserving their traditions in an environment with different values and customs [37,44,48,62]. The narratives reveal a continual apprehension that migration may lead to erosion or complete loss of cultural roots, creating a sense of disconnection with family identity.

##### Coping with New Financial Responsibilities

After migration, many individuals face the financial responsibility of supporting their families, both in the host country and those who remain [20,26,42]. However, sending money to family members in covering daily expenses often leads to challenges, such as paying the bills or feeding their children properly [40]. These experiences reveal the complex dynamics of financial responsibilities that migrants assume, facing the pressure of balancing their personal needs with the well-being of their distant families.

##### Desire to Return

The desire to return home is a constant among migrants, reflecting nostalgia and a connection with their roots, as well as the need to support extended family who remain in the country of origin. Reports of returning are intensified by family issues, such as caring for elderly or ill [48], or from coming of age and the desire to return after university to enable a better future for themselves and their family [24]. These experiences reveal the complexity of the feelings surrounding migration and the desire to return home, which sometimes leads to conflicts between members with different perspectives on the subject.

#### 3.1.3. Strategies for Maintaining Family Functioning

Faced with the repercussions of the cross-border migration process on family life, the migrants employed different strategies to better adapt and integrate into the host country and thus enhance family functioning. These strategies include changing social and family roles, maintaining family ties by increasing interaction and communication between family members, and maintaining cultural and religious practices, through language, religion, and cultural roots.

##### Changes in Social and Family Roles

The migration process often results in significant changes in gender roles within families, influencing both internal dynamics and social expectations. When moving to a new country, many migrants encounter cultural, economic, and legal contexts that differ from those in their societies of origin, leading to changes in the traditional division of responsibilities and family functioning. Men and women may take on different roles in response to new demands, such as the need for both to contribute financially, due to economic precariousness, or to adapt to more advanced gender equality norms. Consequently, many women, previously solely responsible for looking after the household and childcare, now face the challenge of being overwhelmed by multiple roles, accumulating domestic work and financial management [22,26,31,37,47,48,60]. Conversely, many men, accustomed to being the primary providers, are led to engage in domestic and caregiving activities [16,26,35,48].

##### Maintaining Family Ties

One strategy identified for enhancing family functioning involves fostering continuous communication with family members who remain in the country of origin [20,26,29,33,34,35,44,51] and in the household context as well [44,53]. This is especially relevant for maintaining family and cultural connections in situations of migration and geographical separation, and/or maintaining internal ties and cooperation in family life activities and tasks. The utilization of technologies, such as cell phones, social networks, and even online games, has emerged as mechanisms for sustaining these interactions, especially in scenarios where socioeconomic factors and distance limit physical in-person contact [33,35,48].

##### Maintaining Cultural and Religious Habits

Migrants also highlighted a strategy for proper family functioning after the migration process the attempt and challenge of preserving cultural [15,23,36,55,56,58,62], linguistic, and religious habits [53]. Additionally, material gestures, such as sending gifts, were used to strengthen emotional ties to compensate for both physical and cultural distance [26].

## 4. Discussion

This systematic review synthesizes qualitative evidence on the repercussions of cross-border migration on family life, revealing a complex and dynamic process marked by persistent tension between changes and continuity in family functioning. The identified meta-theme—tending between change and maintenance—captures how migrant and refugee families continuously negotiate adaptation in the host society while striving to preserve cultural roots, family identity, and relational bonds. Rather than representing a linear transition, migration emerges as an ongoing process that reshapes family relationships, roles, and meanings over time.

Within this framework, adaptation to a new country unfolds across multiple and often contrasting contexts, which may be either favorable or adverse, exerting significant influence on family functionality and on the experiences of its members [13]. Contexts characterized by xenophobia, social isolation, precarious living conditions, and limited access to formal employment tend to intensify family conflicts and generate negative repercussions for mental health [49,50]. At the same time, migration often reduces shared family time due to long working hours and, in some cases, physical separation, altering daily routines and fostering feelings of homesickness and loss of connection, even when the family migrates together [47]. These conditions frequently require renegotiation of family roles, including women’s increased participation in paid labor alongside expanded domestic responsibilities, as well as men’s engagement in caregiving activities not previously embedded in daily routines [13,20].

Interpreted through the tension between change and maintenance, living in conditions of precariousness emerges as a central factor that destabilizes family functioning. Limited access to basic needs, unstable or informal employment, social isolation, xenophobia, and documentation barriers intersect to produce cumulative disadvantages, heightening stress, weakening support networks, and exacerbating family conflict. Particularly salient are the impacts on mental health, with recurrent reports of loneliness, sadness, and anxiety, frequently linked to family separation, loss of social ties, and prolonged uncertainty [30,53]. These psychological vulnerabilities may also predispose individuals to the use of psychoactive substances and, in more severe cases, to suicidal ideation, intensifying emotional suffering. Such experiences point to the need for interventions that take into account migrants’ prior life trajectories, including traumatic experiences, as well as their cultural values and current demands [48].

These findings reinforce the understanding that migration-related distress is not solely an individual phenomenon but is embedded within family systems and relational contexts. In this sense, family dynamics are particularly sensitive to uneven adaptation processes, in which some members integrate more rapidly into the host culture while others struggle to do so. This asymmetry can generate intergenerational and value-based conflicts, weaken emotional bonds, and reduce the protective role of the family as a source of support, cohesion, and resilience [47,49].

The fear of losing cultural identity further intensifies this tension, especially among families with children born or raised in the host country. Concerns about the erosion of traditions, language, religion, and cultural practices illustrate how integration and cultural maintenance operate as opposing forces within the same family system. This symbolic “tug-of-war” reinforces the importance of spaces and practices that value cultural heritage, as these contribute to emotional well-being, a sense of belonging, and the preservation of family identity in migratory contexts [37].

Financial responsibility represents another critical axis within the change–maintenance framework. Many migrants experience sustained pressure to support two households—one in the host country and another in the country of origin. This obligation is compounded by job insecurity, low wages, and high living costs, leading to financial strain and increased emotional and psychological stress [14]. These pressures frequently intersect with other stressors, reinforcing feelings of instability and vulnerability within family life.

Closely connected to these challenges is the strong and recurrent desire to return to the country of origin. This desire is often driven by emotional obligations, such as caring for elderly parents or ill relatives, as well as by aspirations to raise children within a familiar cultural environment. Economic difficulties, lack of opportunities, financial instability, and a persistent sense of not belonging in the host society further strengthen this inclination to return [64]. Such ambivalence highlights how migratory trajectories are rarely definitive; rather, they are continually re-evaluated in light of family needs, emotional ties, and structural constraints. Similar processes of ongoing reassessment and adjustment are observed in other stressful family situations that require adaptive family functioning, such as living with a chronic health condition [65,66], experiencing the loss of a loved one [67], or navigating palliative care [68].

In response to these cumulative challenges, migrant families actively develop strategies to maintain family functioning. These include adapting social and family roles, strengthening family bonds through sustained communication, and preserving cultural and religious traditions. Although the renegotiation of gender roles may generate tension, it also reflects families’ pragmatic efforts to respond to new economic and social demands. Frequent communication with geographically distant family members, supported by information and communication technologies, plays a crucial role in maintaining emotional connections and transnational family ties [14,52]. Similarly, preserving cultural and religious practices reinforces identity and belonging, functioning as an important source of emotional support and family cohesion, particularly in adverse contexts [14,52].

Taken together, these findings underscore that family functioning in the context of cross-border migration is shaped by a continuous interplay between structural constraints and adaptive strategies, mediated by the enduring tension between change and maintenance. In practical terms, these findings can guide health and social care professionals to assess migrant families beyond individual symptoms, incorporating family relationships, cultural continuity, transnational ties, and socioeconomic stressors into clinical and social assessments, and to design interventions that strengthen family communication, support role negotiation, and mitigate the psychosocial impacts of migration-related adversity.

Furthermore, the importance of public policies and professional practices that recognize the family as a unit of care and that are sensitive to migrants’ previous experiences and cultural backgrounds. Training in multiculturalism, interculturality, and culturally sensitive care is therefore essential for health and social care professionals and students, enabling them to respond more effectively and humanely to the complex needs of migrant families.

Finally, the findings of this systematic review reveal important gaps and directions for future research on refugee and migrant family functioning. Despite advances in understanding migration-related challenges and coping strategies, the literature remains fragmented and predominantly focused on individual experiences, often overlooking family-level and relational processes. Future research should prioritize longitudinal and family-centered designs that examine how family functioning evolves across different stages of the migration trajectory, including pre-migration, transit, settlement, and potential return. Further attention is also needed to the intersections between gender roles, cultural identity, mental health, and socioeconomic conditions, particularly in understudied regions and among marginalized migrant populations. Future systematic reviews would benefit from focusing on intervention studies, policy-driven programs, and comparative analyses across migration contexts, thereby strengthening the evidence base for practices and policies aimed at promoting family resilience and functionality among migrant and refugee populations.

### Limitations and Strengths

Some limitations of the study were the potential exclusion of relevant studies published in languages other than Portuguese, English, and Spanish. Nevertheless, a comprehensive search protocol was employed, which included grey literature databases, leading to an initial collection of 5283 texts. The findings presented are not intended to be generalized and should be interpreted and applied carefully, considering the experiences of each migratory context. This is because qualitative research provides theoretical and contextual insights into the experiences of a limited number of people in specific environments. Furthermore, the heterogeneity of studies conducted across different countries and cultural and socio-economic contexts makes it challenging to generalize the results.

In addition, the findings can be applied to different fields, such as health, psychology, social work, and public policy, with an emphasis on interdisciplinarity and transdisciplinarity. This approach will support the development of cultural integration programs that consider the emotional and cultural needs of migrants and their families. These insights provide a basis for developing more specific psychological and social support programs focused on family needs.

## 5. Conclusions

This systematic review with meta-synthesis identified that families who experience the cross-border migration process encounter different possible contexts in the settlement. Many of them reveal that they live in the underworld, where experiences of the precariousness of life in the tangled cultural context are constant. In contrast, some individuals, reflecting on their experiences in their country of origin, feel lucky, as they can perceive a new life with more security, the possibility of work and housing, and a higher quality.

Faced with various contexts, families experience repercussions and challenges, including heightened family conflicts, coping with mental health repercussions, family separation, loss of ties and homesickness, reduced family life due to long working hours, use of psychoactive substances, fear of losing cultural roots, new financial responsibilities and the constant desire to return to their country of origin.

All these confrontations and repercussions lead migrant families to resort to strategies to preserve their functioning, such as adjusting their social and family roles and maintaining family ties and cultural/religious habits.

## Figures and Tables

**Table 1:** Summary Table of the Characteristics of the Selected Studies.

Author (Year)	Country	Participants	Data Collection Method	Analysis Method	QARI (%)
Syam et al., 2019 [13]	Lebanon	28 migrants	In-depth interview	Thematic content analysis	90
Kerlaff, 2023 [14]	United Kingdom	21 parents and 8 children	In-depth interview	Interpretative phenomenology	80
Farr et al., 2018 [15]	United Kingdom	4 migrant mothers	Interview	Interpretative phenomenology	90
Wali; Renzaho, 2018 [16]	Australia	164 migrants	Focus groups	Thematic analysis	90
Lim, 2009 [20]	United States	21 adult migrants	In-depth interview	Phenomenology	80
Patel et al., 2021 [21]	United States	42 migrant teenagers	Community participation method and in-depth interviews	Thematic analysis	80
Babatunde-Sowole et al., 2016 [22]	Australia	20 migrant women	Interview	Thematic analysis	80
Selleck, 2023 [23]	United Kingdom	24 mothers and children	Participant observations and ethnographic conversations	Ethnography	80
Ramírez Varela, 2022 [24]	Chile	18 migrant teenagers	Group interviews	Content analysis	90
Patel et al., 2016 [25]	United States	189 migrant students	Semi-structured interview	Content analysis	80
Restrepo et al., 2019 [26]	Colombia	13 migrants	Interviews	Interpretative hermeneutics	90
Ayika; Duna; Firdau; Mapedzaham, 2018 [27]	Australia	164 migrants	Participatory action research and focus group	Thematic analysis	80
Orjuela- Grimm et al., 2022 [28]	United States	46 teenagers	Semi-structured interview	Thematic analysis	80
Liddell et al., 2022 [29]	Australia	13 refugees	Semi-structured interview	Thematic analysis	90
Pittaway; Riggs; Dantas, 2023 [30]	Australia	23 participants	Semi-structured interview	Thematic analysis	90
Kok; Khor; Hon; van Schalkwyk, 2021 [31]	Malaysia	25 refugee children	Verbal and non-verbal narratives (storytelling)	Thematic analysis	90
Weitzman et al., 2024 [32]	Costa Rica	33 mothers	In-depth interviews	Thematic analysis	70
Zapata-Martínez, 2020 [33]	Chile	13 families- 27 interviewees	In-depth interview	Phenomenology	70
Ryan et al., 2009 [34]	United Kingdom	15 men; 15 women	Focus groups and interviews	Content analysis	70
Graham et al., 2012 [35]	Indonesia and the Philippines	32 children; 50 caregivers	Semi-structured interview	Thematic analysis	80
Schuler et al., 2022 [36]	Brazil and Switzerland	4 grandparents; 4 mothers; 4 grandchildren	Life story—semi-structured interview	Thematic Content Analysis	80
Ashbourne et al., 2021 [37]	Canada	33 Arab refugees	Semi-structured interview	Thematic analysis	100
Zhao et al., 2018 [38]	China	25 children; 17 parents; 13 grandparents; 24 key informants	In-depth interviews	Data-driven theory	80
Godin; Sigona, 2025 [39]	United Kingdom	37 interviews	In-depth interviews	Coding	100
Eid; Diah, 2019 [40]	Malaysia	30 families: 9 women 21 men	In-depth interviews	Thematic analysis	100
Critelli et al., 2021 [41]	Kyrgyzstan	20 individuals	In-depth interviews	Consensual Qualitative Methodology	90
Sassone; Lapenda, 2019 [42]	Argentina	60 migrants	In-depth interviews	Thematic analysis	80
Hernández, 2013 [43]	United States	30 migrant teenagers	Longitudinal qualitative study with interviews	Coding strategies derived from open coding	100
Mehrotra; Calasanti, 2010 [44]	United States	38 migrants	In-depth interviews	Thematic analysis	100
Eltanamly et al., 2022 [45]	The Netherlands	27 parents from 16 families	In-depth interviews	Data-driven theory	70
Schmidt; Bhuyan; Lash, 2022 [46]	Canada	35 migrant women	Interviews and field diary	Iterative approach	80
Bergnehr, 2022 [47]	Sweden	10 parents	Interviews and field notes	Narrative analysis	80
Iyakaremye; Musabyiman; Umutoni, 2019 [48]	Africa	21 men	Interviews and focus groups	Content analysis	80
Rapaport; Doucerain, 2021 [49]	Canada	20 migrants (couples)	Life story interview	Thematic and dyadic analysis	100
Sim et al., 2023 [50]	Canada	40 migrants	Interviews	Inductive thematic analysis	90
Ryan, 2011 [51]	United Kingdom	46 migrants	Focus groups and interviews	Narrative analysis	90
Phoenix, 2023 [52]	United Kingdom	53 participants (38 women and 13 men)	Interview	Thematic and narrative analysis	80
Gangamma, 2018 [53]	United States	11 migrants	Interviews	Phenomenology	90
Muruthi et al., 2020 [54]	United States	14 refugees	Interviews	Ethnography	80
Frounfelker et al., 2017 [55]	United States	81 refugees	Focus groups	Thematic analysis	80
Umubyeyi; Mtapuri, 2019 [56]	South Africa	16 migrants	Interviews	Interpretive analysis	80
McCleary et al., 2020 [57]	United States	36 refugees	Focus groups	Ethnocultural method of thematic categories	100
Renzaho; Green; Mellor; Swinburn, 2011 [58]	Australia	85 migrants	Focus groups	Thematic analysis	100
McCleary, 2017 [59]	United States	36 refugees	Focus groups and interviews	Thematic analysis	100
Yaqun Li et al., 2024 [60]	Portugal	25 migrants	Interviews and observations	Thematic analysis	80
Zharkynbekova et al., 2024 [61]	Kazakhstan	30 migrants	Interviews	Employing discourse analysis	80
Ryan et al., 2024 [62]	United Kingdom	5 Afghan women	Longitudinal qualitative data	Case study analysis	100
García et al., 2024 [63]	United States	70 participants	Semi-structured interviews	Content analysis	100

Source: Authors’ data collection.

**Table 2:** Primary data aggregation process—Maringá, PR, Brazil, 2025.

Findings	Sub-Themes	Themes	Meta-Theme
Our whole house is full of rats… my little girl is less than a year and a half old and she ate the rat droppings and got sick with vomiting and diarrhea…If only someone would help me… I cry, beg, and plead, but no one is willing to help me [migrant] [50] In our home country, we had our traditions and culture, and there we had rules [for how children should behave]. But here, everything has changed. Here, they [the children] mix and adjust to new traditions and cultures -Somali, Afghan, Swedish, all kinds. Everyone tries to teach them about their culture, and then they [the children] receive another culture. [Jacob-migrant] [47] If I’d had my family around, everything could have been easier. Six years without living with my family. But I’m thinking about my children’s future. My daughter was only 5 months old when I arrived here, now she’s 7. My 1-year-old son is now 8, so I’m just fighting for their future. I could even have gone to study because I believed I was intelligent enough. But since I had no support, no relatives, nothing, I had to take care of myself, stop studying, and go to work. Pay the bills, put food on the table”. (Participant 32) [29] Isolation is the main problem. If you are not working, then you are isolated, if you don’t have a big support from your relatives, friends, and family, then you are isolated. So, whether you have relatives, friends, a big circle or you are working, otherwise you are just isolated. (Indian participant, FGD 8) [16]	Living in the underworld: experiences of xenophobia, isolation, and the precariousness of life in a tangled cultural context	Living in multiple possible contexts: space and relationships influencing family functionality	Tension between change and maintenance: the search for family functionality in the face of cross-border migration
Many family members and friends call me to ask how I managed to leave Gaza and demand that I make it easier for them to come. They always complain about the harsh living conditions, especially power and water cuts, unemployment, border closures and working without pay. Some of them lost their homes as a result of the last war in Gaza and had to live in shelters like the destitute. With all these challenges, they told me how incidents of crime and suicide are on the rise in Gaza society, especially among young people. When they tell me about their constant suffering, I am more motivated to stay in Malaysia until I reach my goal of resettling in a third country. (Majid migrant) [39] I am honored to be part of this great society (the United States). And (pause), at least, they help me a lot. They make me live safely here. [Iraqi refugee] [53] I’m lucky, we have a whole apartment to ourselves. My husband… He is a plumber and has a good trade, so he earns enough money… we can certainly afford a lot more than in Poland because I could never afford to rent a three-bedroom apartment in Poland, I have two cars. (Kinga, focus group with young Polish mothers) [34] After my family arrived, we felt better, we felt safer […] (Male godfather, Heydari family) [14]	Feeling lucky to experience life with security, work, housing, and quality		
My husband once held three chairs and broke them on me. He hit me with a cane made of whatever… Once he hit me with a hookah. He does this because he knows how weak I am… here I have nowhere to go and no one to turn to.” (woman, 19) [13] It happened a lot like my mother left my father, he couldn’t get a job, and she has money now, because of the fights they split up. (Young female) [30]	Potentiating family conflicts	Challenges faced and repercussions on family life after migration	
Sometimes I wish I could die to get rid of this life we’re living. My husband is tired too. He always tells me that it’s better for him if he dies or if a car runs him over”. (woman, 32) [13] At the beginning [of our separation], if I cried there, my daughter cried here. My mother-in-law kept telling me ‘When you call, don’t cry! Try to get over it!’ I tried not to cry, but you miss her and worry. I cried anyway, most of the time. Now and then I got stressed. Then I had an outburst of anger. [migrant mother] [41]	Repercussions on Mental Health		
I felt I was leaving a part of myself behind because my family, cousins, and friends are all there. I have known since I was little. I had to leave all that. It was challenging for me… I wanted to still be with them. (an 18-year-old girl from Haiti) [25] I think the separation of so many years… has left wounds in his heart. And maybe he doesn’t express it, and here we are. Sometimes his attitude is sometimes… a bit rude, which hurts me. For example, his answers. Sometimes I don’t feel he wants me as his mother, even though he tries. [Gabriela—migrant] [46] The longing for my Brazilian family always presses down on my chest… and the absence of my family here with me… it is really difficult” (Patrícia—migrant mother) [36]	Family separation, loss of ties, and homesickness		
It is extremely difficult for children because you can’t look after them, raise them, or keep an eye on them [in the same way] when you only see them two or three hours before bedtime. You barely have time to ask about their homework. And sometimes I work until 9 p.m. or 11 p.m., and they’re in bed when I get home… [Jacob-migrant] [47] I’ve just given birth on my own, and I have to do everything, again I’m responsible for the house and the children. I have to get up early to prepare the children’s snacks and look after the house. I was in pain after giving birth, I had a tear and it hurt a lot. And he, he just went out to work. That’s it! [migrant] [49]	Reduced family life		
(…) The big difference is that where women earn more, the whole family benefits from that income, which is used for household needs. In contrast, when men earn a lot of money, they use it to consume a large amount of alcohol with their male colleagues, leaving only a small amount of their earnings for the rest of the family members. [refugee] [48]	Use of psychoactive substances		
As for my teenage daughter, you don’t know what will happen. She might meet someone and as you know, we’re Muslims and our customs and traditions are completely different. [P25-Feminine] [37]	Fear of losing cultural roots		
We have to feed ourselves and organize ourselves, keep things together, and then what I do is send him (father) to help him settle down more or less as he did before because things don’t work out there, it’s a bit forced. (Ada, 23 years) [26]	Coping with new financial responsibilities		
I see myself studying in the future and then going back to my country”. (E01M, Peru) [24] I told my daughter I’m going back next year. I don’t know what she’s going to do, I hear there are nurseries… [her Granddaughter] is going to be four, she’s going to the nursery… but I can’t keep sitting here, because my sister is very ill… she has cancer. (Karolina) [34]	Desire to return		
Here I’m a man and a woman. If the children are sick, I have to take them for medical treatment, when they call from school, I have to go. I have to pick up the food, I have to cook and clean… I’m responsible for everything. I feel for my husband because he works all day and comes home at night, but he still can’t give me everything I need” (Woman, 44) [13] That was the most difficult thing I saw here… it was the first time I changed a diaper, so it was difficult for me because I kept telling myself that this is not my role.” (African male participant, FGD 4) [16] I have four younger brothers and sisters, like babies, and now my mother goes out and leaves me to look after them. She goes to friends, she goes to big community parties, and she just thinks I should do it. I have to clean and cook for them. If my dad was around, he’d make her do it, now he’s not here, she doesn’t care and I have to do it. This happens to a lot of my friends too”. (Young female) [30]	Changes in social and family roles	Strategies for maintaining family functioning	
It is very important [to be connected] because there they have a lot of problems… food, security, diseases, and fights going on all the time. You don’t know. Today you can talk to them and tomorrow there will be fighting and your relative will be killed. You always worry, so you keep in touch: “Are you okay? Ah, okay, good!” (Participant separated from family for 20 years) [20] Through PlayStation you can also send text messages and talk on camera, sometimes they communicate through Facebook, or they communicate through Play, and that’s where we talk. PlayStation has chat -yes, that’s right-, as well as playing games, you can download the YouTube program, you can download Skype (if you have play with a camera) and talk to them on Skype. You can watch Blu-ray movies, you can watch DVDs, all of that. (Father 35, Cali) [33]	Maintaining family ties		
I want them to know and be proud of where their mother comes from, and you know, have that contact with the Spanish family, just to keep the roots […] Spanish and I am not going to change that, so yes, I want my children to know where they come from […] Apart from not being able to communicate with my family, it’s my heritage, it’s my language, I don’t want them to be separated from me, I want them to know that they’re also half Spanish. (Bianca) [15] So, the sort of saving grace for me was that there was a church that we went to that was worshipped in the same way and the language was the same. And the people were similar, so, you know, I think that’s, that was the will, kept me from absolutely (laughs) falling apart probably. [Lizzie—migrant] [52]	Maintaining cultural and religious habits		

Source: Authors’ data collection and analysis.

## Data Availability

The articles and extraction tables are available in the body of the article in its tables and references.

## Supplementary Materials

The following supporting information can be downloaded at: https://www.mdpi.com/article/10.3390/ijerph23020165/s1, Table S1: Complete search strategy carried out for the databases, 2025, File S1: PRISMA_2020_checklist.
