# Supplementary material for: Repercussions of the Cross-Border Migration Process on Family Life: Systematic Review with Meta-Synthesis
Source: Int J Environ Res Public Health. 2026 Jan 28;23(2):165. doi: 10.3390/ijerph23020165 (PMC12940735; doi:10.3390/ijerph23020165)
Supplement: Supplementary file 1 [file ijerph-23-00165-s001.zip › ijerph-4058571-supplementary-1.pdf]

Supplementary Material Table S1 - Complete search strategy carried out for the databases, 2025.

Database	Search strategies	Additional details
PubMed	(("Refugees" [Title/Abstract] OR "Migrants" [Title/Abstract] OR "Transients and Migrants" [Title/Abstract] OR "Emigration and Immigration" [Title/Abstract]) AND ("family relations" [Title/Abstract] OR "family" [Title/Abstract] OR "family nursing" [Title/Abstract] OR "family separation" [Title/Abstract]) AND ("qualitative studies" [Title/Abstract] OR "qualitative" [Title/Abstract] OR "Perception" [Title/Abstract]))	Filters applied: 01/01/2009 to 31/12/2024; languages: Portuguese, English and Spanish; *Full text*.
LILACS	(("Refugees" OR "Migrants" OR "Transients and Migrants" OR "Emigration and Immigration" AND "family relations" OR "family" OR "family nursing" OR "family separation" AND "qualitative studies" OR "qualitative" OR "Perception"))	Filters applied: 2009 to 2024; languages: Portuguese, English and Spanish.
PsycInfo	(("Refugees" OR "Migrants" OR "Transients and Migrants" OR "Emigration and Immigration" AND "family relations" OR "family" OR "family nursing" OR "family separation" AND "qualitative studies" OR "qualitative" OR "Perception"))	Filters applied: 2009 to 2024; *Journal article* and *Magazine article*.
SCOPUS	TITLE-ABS-KEY (((*"refugees"* OR *"migrants"* OR *"transients and migrants"*) AND (*"family relations"* OR *"family"* OR *"family nursing"* OR *"family separation"*) AND (*"qualitative"* OR *"studies" OR "qualitative" OR "perception"*)))	AND LIMIT-TO OR (LIMIT-TO (PUBYEAR, *2024*) OR (PUBYEAR, *2023*) OR (LIMIT-TO (PUBYEAR, *2022*) OR LIMIT-TO (PUBYEAR, *2021*) OR LIMIT-TO (PUBYEAR, *2020*) OR LIMIT-TO (PUBYEAR, *2019*) OR LIMIT-TO (PUBYEAR, *2018*) OR LIMIT-TO (PUBYEAR, *2017*) OR LIMIT- TO (PUBYEAR, *2016*) OR LIMIT-TO (PUBYEAR, *2015*) OR LIMIT-TO (PUBYEAR, *2014*) OR LIMIT-TO (PUBYEAR, *2013*) OR LIMIT-TO (PUBYEAR, *2012*) OR LIMIT-TO (PUBYEAR, *2011*) OR LIMIT-TO (PUBYEAR, *2010*) OR LIMIT- TO (PUBYEAR, *2009*) AND (LIMIT-TO (DOCTYPE, *"ar"*)) AND (LIMIT-TO (LANGUAGE, *"English"*) OR LIMIT-TO (LANGUAGE, *"Spanish"*) OR LIMIT-TO (LANGUAGE , *"Portuguese"*)).
Web of Science	(("Refugees" OR "Migrants" OR "Transients and Migrants" OR "Emigration and Immigration") AND ("family relations" OR "family" OR "family nursing" OR "family separation") AND ("qualitative studies" OR "qualitative" OR "Perception"))	Sort by: most relevant first
CINAHL	((MH "Refugees" OR MH "Migrants" OR MH "Emigration and Immigration") AND (MH "Family Relations" OR MH "Family Nursing") AND (TX "qualitative" OR TX "perception")) AND (LA "English" OR LA "Portuguese" OR LA "Spanish") AND (DT 2009-2024)	Filters applied: 2009 to 2024; languages: Portuguese, English and Spanish.

Social Science Citation Index	(("Refugees" OR "Migrants" OR "Transients and Migrants" OR “Emigration and Immigration”) AND ("family relations" OR "family" OR "family nursing" OR “family separation”) AND ("qualitative studies" OR "qualitative" OR "Perception"))	Sort by: most relevant first
Networked Digital Library of Theses and Dissertations	(("Refugees" OR "Migrants" OR "Transients and Migrants" OR “Emigration and Immigration”) AND ("family relations" OR "family" OR "family nursing" OR “family separation”) AND ("qualitative studies" OR "qualitative" OR "Perception"))	No filters
New York Academy of Medicine Grey Literature Report	(("Refugees" OR "Migrants" OR "Transients and Migrants" OR “Emigration and Immigration”) AND ("family relations" OR "family" OR "family nursing" OR “family separation”) AND ("qualitative studies" OR "qualitative" OR "Perception"))	No filters
CAPES dissertation and thesis database	(("Refugees" OR "Migrants" OR "Transients and Migrants" OR “Emigration and Immigration”) AND ("family relations" OR "family" OR "family nursing" OR “family separation”) AND ("qualitative studies" OR "qualitative" OR "Perception"))	No filters
Index to Theses	(("Refugees" OR "Migrants" OR "Transients and Migrants" OR “Emigration and Immigration”) AND ("family relations" OR "family" OR "family nursing" OR “family separation”) AND ("qualitative studies" OR "qualitative" OR "Perception"))	No filters

Source: Prepared by the authors, 2025.
